# Progress in Circulating Tumor Cell Research Using Microfluidic Devices

**DOI:** 10.3390/mi9070353

**Published:** 2018-07-14

**Authors:** Hogyeong Gwak, Junmoo Kim, Leila Kashefi-Kheyrabadi, Bongseop Kwak, Kyung-A Hyun, Hyo-Il Jung

**Affiliations:** 1 School of Mechanical Engineering, Yonsei University, 50 Yonsei-ro, Seodaemun-gu, Seoul 03722, Korea; kirapi83@gmail.com (H.G.); o2akira@naver.com (J.K.); kashefi.leila@gmail.com (L.K.-K.); 2 Daegu Research Center for Medical Devices and Rehab., Korea Institute of Machinery and Materials. Engineering, 330 Techno Sunhwan-ro, Yuga-myeon, Dalsung-gun, Daegu 42994, Korea; bsk@kimm.re.kr

**Keywords:** circulating tumor cell (CTC), cancer, microfluidic device, CTC isolation, CTC analysis

## Abstract

Circulating tumor cells (CTCs) are a popular topic in cancer research because they can be obtained by liquid biopsy, a minimally invasive procedure with more sample accessibility than tissue biopsy, to monitor a patient’s condition. Over the past decades, CTC research has covered a wide variety of topics such as enumeration, profiling, and correlation between CTC number and patient overall survival. It is important to isolate and enrich CTCs before performing CTC analysis because CTCs in the blood stream are very rare (0–10 CTCs/mL of blood). Among the various approaches to separating CTCs, here, we review the research trends in the isolation and analysis of CTCs using microfluidics. Microfluidics provides many attractive advantages for CTC studies such as continuous sample processing to reduce target cell loss and easy integration of various functions into a chip, making “do-everything-on-a-chip” possible. However, tumor cells obtained from different sites within a tumor exhibit heterogenetic features. Thus, heterogeneous CTC profiling should be conducted at a single-cell level after isolation to guide the optimal therapeutic path. We describe the studies on single-CTC analysis based on microfluidic devices. Additionally, as a critical concern in CTC studies, we explain the use of CTCs in cancer research, despite their rarity and heterogeneity, compared with other currently emerging circulating biomarkers, including exosomes and cell-free DNA (cfDNA). Finally, the commercialization of products for CTC separation and analysis is discussed.

## 1. Introduction

Tumor cells form a three-dimensional shape and send signals to the nearby blood vessel network to form new blood networks near themselves in a process known as angiogenesis. Because of angiogenesis, the blood vessel network near a tumor is extremely developed, and high levels of nutrient delivery and gas/waste exchange occur. Despite the well-developed blood network in the tumor microenvironment, the tumor cells experience starvation and suffocation because of their fast metabolic activity, very high cell packing density, and infinite proliferation. The tumor cells begin to experience stress and separate as individual cells from the main tumor body. These individualized tumor cells move toward the blood cell network and digest the extracellular matrix using a collagenase such as matrix metalloproteinase. The individualized tumor cells reach the pericyte and make a small hole for intravasation. A tumor cell floating in the blood vessel network is known as a circulating tumor cell (CTC) [1].

CTCs in human blood vessels represent one of the main causes of recurrent or metastatic cancer. However, a very small number of CTCs (1–1000/mL) are found in human blood, which also contains large numbers of erythrocytes (~5 × 10^9^/mL), leukocytes (~4 × 10^6^/mL), and platelets (~3 × 10^8^/mL). Moreover, not all the CTCs are in a ready state for recurrence or metastasis. The tumor cells are continuously changing their characteristics through epithelial-mesenchymal transition (EMT) or mesenchymal-epithelial transition (MET) [2]. Because of the rarity and heterogeneity of CTCs, the detection of CTCs is not easy and remains a formidable challenge for clinical use.

Currently, the CellSearch^®^ system (Menarini Silicon Biosystems, Inc., Bologna, Italy) is the only US Food and Drug Administration (FDA) approved CTC detecting system, and it is an epithelial cell adhesion molecule-based detecting system. The CellSearch^®^ system can be used for patients with metastatic breast, prostate, or colorectal cancer to make a prognosis of tumor recurrence or metastasis. Since the introduction of the CellSearch system in 2004, many researchers have studied the relationship between the number of CTCs and the survival rate [3]. This is a powerful system for clinical application, but it has a comparably low detecting accuracy and is not able to distinguish between heterogenic tumor cell types.

The microfluidic approaches are usually more cost-effective than batch approaches. This is because they can handle a very low volume of reagent (such as an antibody and magnetic nanoparticles) and because they can deal with the considerable volume of samples obtained in a continuous manner as needed [4]. In addition, because of the ease of multi-disciplinary and intelligent integration, which is one of the advantages of microfluidics, many experimental steps performed on a laboratory scale can be implemented using a single chip. This not only avoids the loss of rare CTCs caused by replacing tubes or tips during multiple experimental steps but also makes the process of the experiment more convenient to the user through automation. Thus, many researchers have tried to develop microfluidic system-based CTC-detecting methods using the unique properties of CTCs such as density [5], size [6], deformability [7], and differences in membrane protein expression [8] in the past decades.

In this paper, we review the trends in CTC research focused on microfluidic approaches. We classify CTC enrichment methods into four types and compare these methods with five performance categories. In addition, the results of CTC analysis for the next steps in cancer research after CTC isolation are investigated. The critical concerns regarding CTCs are discussed in terms of the importance of studying CTCs compared to other circulating biomarkers and the commercialization of CTC separation and analysis equipment.

## 2. Trends in Circulating Tumor Cell (CTC) Research

**Figure 1:**
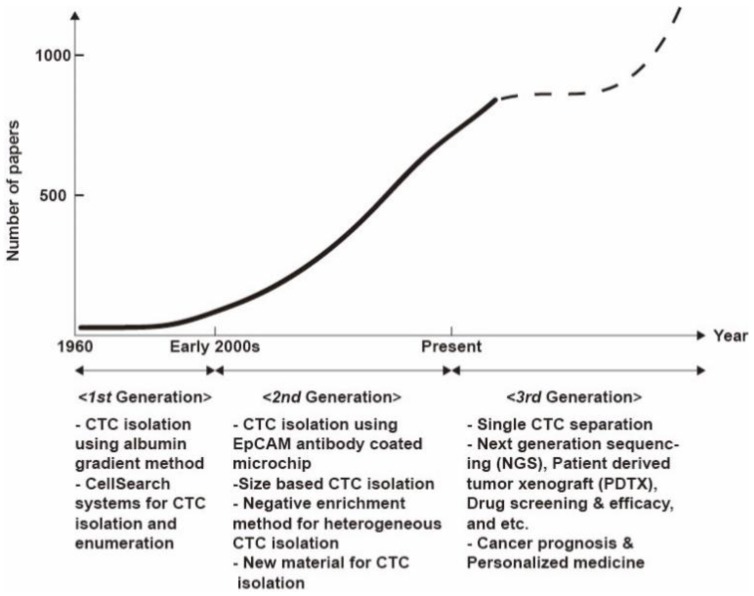
Schematic sketch of research trends in circulating tumor cells (CTC) isolation. The solid line shows the number of papers according to the time (http://www.scopus.com), and the dashed line is a prediction of the number of articles published in the future.

**Figure 2:**
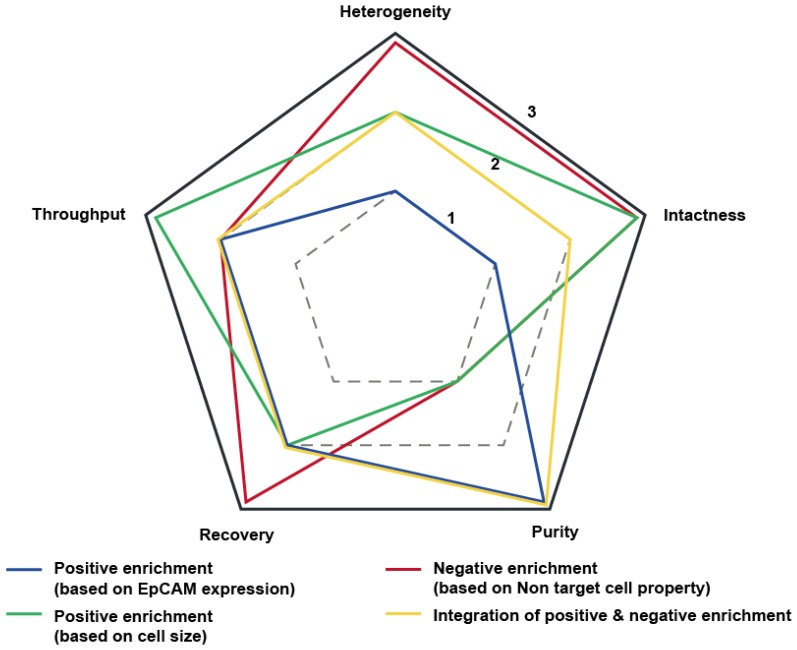
Schematic sketch of the comparison of different methods for CTC isolation. Five performance categories are used for the comparison: (1) heterogeneity of isolated CTCs, (2) intactness of isolated CTCs, (3) purity, (4) target cell recovery, and (5) throughput. A scale of 1 to 3 was used to rank each category, where 3 represents the highest score.

Since cancer cells were initially found in 1869 in the blood of patients who died from breast cancer [9], studies have been conducted to isolate these cancer cells from blood and to analyze them to understand cancer metastasis. There have been a large number of studies reporting on CTC research (Keywords: “circulating tumor cell”, “isolation”, “separation”, “enrichment”, “purification”, “analysis”, “characterization”, and “detection”; http://www.scopus.com). We plotted the trend of CTC research based on the number of papers over time (Figure 1). And we classified the CTC separation based on batch processing like gradient methods and CellSearch^®^ system as a first generation, and classified based on microfluidics as a second generation. As a third generation, we carefully speculate that isolated single CTC analysis, cancer prognosis, and personalized medicine will lead the field of CTC research. The epithelial cell adhesion molecule (EpCAM), which is involved in intercellular adhesion of epithelial cells and signal transduction, is frequently overexpressed in primary and metastatic cancer. Thus, CTCs were presumed to express EpCAM on their surface because of their epithelial origin. In 2004, CellSearch, a technology for CTC separation using magnetic nanoparticles coated with antibodies that specifically bind to EpCAM, was approved by the FDA. This has provided an opportunity for many researchers to work with separated CTCs, and CTC analyses based on this system have greatly increased in popularity. The results from clinical studies have indicated that the overall and progression-free-survival (OS, PFS) rates of cancer patients are related to the number of CTCs in the blood [10]. Over the past decade, numerous papers have been published on methods for EpCAM-based CTC separation in microfluidic devices. The EpCAM antibody is immobilized on the surface of a microfluidic channel to capture the CTCs as blood passes through the microfluidic device. This method results in a high purity, but the throughput is limited to a low shear force compared to the net reaction force between antigens on CTCs and antibodies immobilized on the channel surface. A separation method using magnetic particles with an EpCAM antibody was proposed as an alternative method to solve the throughput issue, but it has a problem involving the contamination of magnetic particles. Moreover, it has been reported that CTCs with down-regulated levels of EpCAM expression are present due to the EMT [11]. EpCAM-independent CTC isolation has been studied using the fact that epithelial-originated cancer cells are usually larger than hematological cells [12]. Depending on whether external force is used except for fluid delivery, there are passive separation methods such as filtration and inertial force-based separation and active separation methods such as acoustophoresis and dielectrophoresis [13]. Although size-based separation can separate CTCs with varying levels of surface protein expression, the presence of small CTCs, equal to or smaller than leukocytes, presents as a hurdle for high-purity separation [14]. Some studies have suggested that small CTCs may play an important role in metastatic cancer [15]. Therefore, a negative enrichment technique that separates intact CTCs by specifically removing the blood cells has been developed [16]. The method of separating heterogeneous CTCs using magnetic particles coated with leukocyte-specific antibodies can solve both of the above-mentioned throughput and magnetic particle contamination problems. Studies have also been carried out to isolate high-purity CTCs through the combination of leukocyte-specific antibodies [17]. In addition to these mainstream studies, studies are underway to improve separation efficiency using various materials or to release CTCs bound to antibodies [18]. Recently, studies on the isolation of CTC clusters, which are composed of two or several aggregated CTCs, and platelet-covered CTCs have emerged [19,20]. There is growing evidence to indicate that CTC clusters promote mutual survival in the bloodstream and, consequently, improve metastatic ability. The CTCs associate with platelets to escape from the innate immune system and oxidative stress. In addition to enrichment studies on CTCs, application studies such as the analysis of isolated CTCs at the single-cell level have been carried out [21]. One of the research topics related to single-cell analysis is to identify determinants of metastasis by profiling individual CTCs because only a few cells among the enriched CTCs have tumor-initiating properties. After the maturation of the above-mentioned technologies, it is expected that research on customized medical therapies will be conducted using pure, high-concentration CTCs in the future. Figure 2 shows the performance evaluation of the above-mentioned methods for CTC isolation according to five categories: (1) heterogeneity of CTCs, (2) intactness of isolated cells, (3) purity, (4) recovery of target cells, and (5) throughput of sample. The heterogeneity such as different cell sizes and various protein expression levels on the surface is the inherent characteristic of cancer cells. The intactness of isolated cells refers to the intact state of cells that have not undergone any treatment, such as conjugation of antibodies for separation. Purity and recovery are defined as the number of isolated CTCs divided by the total number of isolated cells and the number of isolated cells relative to the initial number of cells, respectively. Throughput means the maximum rate (volume per time or number per time) for the whole process to be complete. Below, we discuss the detailed principles and performance of microfluidic devices based on recent studies.

### 2.1. Positive Enrichment of CTCs Using Antigen-Antibody Reaction

**Figure 3:**
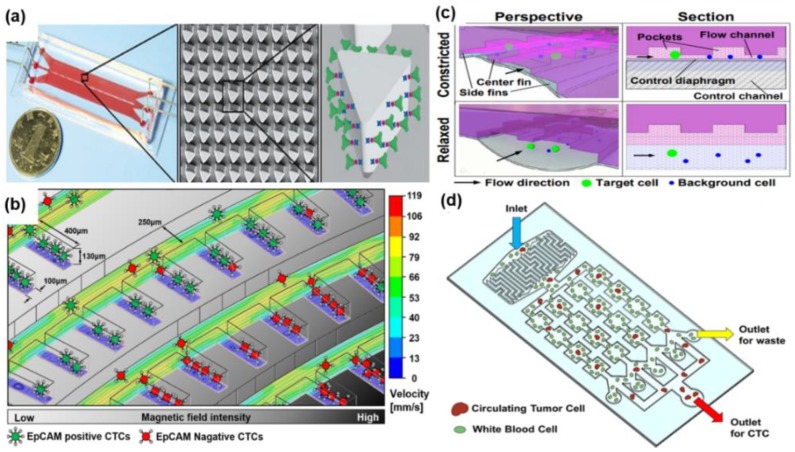
Positive enrichment of CTCs. (**a**) Antibody-coated triangular microarray structures of the SDI-Chip. The surface of triangular microarray structures is coated by epithelial cell adhesion molecule (EpCAM) antibodies. CTCs are selectively captured depending on EpCAM expression level since the rotated triangular micro-pillars makes different hydrodynamic gradient shear forces; (**b**) The spiral shape of the microfluidic device for the selective isolation of CTCs depending on the EpCAM expression level. EpCAM positive CTCs bind more magnetic particles than EpCAM negative CTCs. EpCAM positive CTCs are captured far away from the magnet due to the magnetic force different; (**c**) CTCs and non-target cells move into the flow channel and CTCs are captured in the pockets by moving the diaphragm upwards. CTCs are released by moving the diaphragm downward after passing through non-target cells; (**d**) The p-MOFF device for label-free separation of CTCs from blood cells. CTCs and white blood cells (WBC) pass through the multi orifice channels. Larger CTCs than WBC are separated by hydrodynamic force. Reproduced from ref. [22,25,26,27] with permission from 2017 John Wiley & Sons, Inc. (Hoboken, NJ, USA), 2015 Royal Society of Chemistry, 2018 ELSEVIER, and 2016 Impact Journals.

The positive enrichment method refers to a method for specifically separating a target cell using the characteristics of the target cell, such as surface biomarker expression and size. A size-dictated immunocapture chip (SDI-chip) based on deterministic lateral displacement (DLD) was developed by Ahmed et al. to selectively enrich and detect CTCs (Figure 3a) [22]. Triangular microarray structures are situated in the middle of the SDI-chip. Hydrodynamic gradient shear forces are generated around the rotated triangular micro-pillars. In order to selectively capture the CTCs the surface inside the chip was immobilized by an antibody. CTCs are captured at different positions on the micro-pillars according to the EpCAM expression levels. CTCs with high EpCAM expression levels are captured in regions with relatively high shear stress, and CTCs with low EpCAM expression levels are captured in low shear stress regions. The capture efficiency and purity of CTCs were reported to be approximately 92.2% and 82.3%. With this SDI-Chip, the different distribution of CTCs around the micro-pillars due to the differences in EpCAM expression levels enabled a high capture efficiency and purity. However, the throughput is low for methods in which antibodies are coated on the surface. The binding force between antibody and CTCs should be higher than the CTC migration force in the fluid in order to capture CTCs. Antibody-coated magnetic particles have been used to solve these problems. Chang et al. developed a parallel flow micro-aperture chip system [23]. Antibody-coated magnetic particles were conjugated with CTCs before the injection of the blood sample. Magnetic particles with CTCs and free magnetic particles are captured by a magnet. The capture yield of CTCs was reported to be about 95%. Many free magnetic particles remain after capturing CTCs. A second magnet and aperture structure are used to remove the free magnetic particles. The aperture structure and swinging the second magnet on top of the chamber move the free magnetic particles down through the apertures and increase the purity of the CTCs. Then, the captured CTCs are analyzed using immunofluorescence. There are many differences between metastatic and non-metastatic cancer cells, and one of them is the EpCAM expression level [24]. The parallel flow micro-aperture chip cannot separate heterogenic CTCs with different protein expression levels. Thus, Kwak et al. developed a microfluidic device with a spiral shape to selectively isolate heterogenic CTCs according to EpCAM expression levels (Figure 3b) [25]. Two different breast cancer cell lines (MCF-7 and MDA-MB-231 cells) were used to confirm the selective isolation of CTCs. EpCAM antibody-conjugated magnetic nanoparticles were mixed with CTCs. CTCs with a high EpCAM expression level conjugated more with the magnetic nanoparticles and were captured first in the channel by the high magnetic force than CTCs with a low EpCAM level. The average selectivity and the purity of EpCAM-positive (MCF-7) and EpCAM-negative CTCs (MDA-MB-231) were reported as 6.1:4.8 and 96.3% and 81.2%, respectively. The heterogenic CTCs were selectively and simultaneously isolated with a high capturing efficiency. These positive enrichments specifically capture CTCs using their surface protein expression. These positive enrichments specifically can be used to capture CTCs using their surface protein expression, but most positive enrichment methods cannot completely isolate heterogenic CTCs because some CTCs have no EpCAM expression.

### 2.2. Positive Enrichment of CTCs Based on Size

CTCs are tumor-derived epithelial cells that are the origin of metastatic disease, and CTCs are usually larger than blood cells [17,28]. A size-based microfluidic device that consists of a triangular pillar (the bulk filtration area) and filter channel array (single cell filtration area) was designed by Gao et al. [29]. Blood cells pass through the bulk filtration area. CTCs remain in the main channels, and other cells (White blood cell, WBC; Red blood cell, RBC) pass through the main channels to the side channels via the filter in the single cell filtration area. The sensitivity, specificity, and capture efficiency of these microfluidic devices are 82.7%, 100%, and 94%, respectively. This chip design as a single chip makes it easy to process blood samples, from blood cell elimination to single CTC capture, in one step. However, this filtration method has a problem with channel clogging, which means that this device cannot handle a large volume of samples. Another filtration device is the resettable cell trap, which is a two-layer polydimethylsiloxane (PDMS) structure comprising a resettable cell trap channel (a sample-carrying upper flow channel) and a diaphragm control channel (a lower fluid-filled control channel) (Figure 3c) [26]. CTCs and other cells move into the resettable cell trap channel, and then, CTCs are captured in pockets and other cells pass through when the diaphragm moves up the resettable cell trap channel. In order to prevent clogging problems, the resettable cell trap chip uses a diaphragm controlled by pressure. CTCs can be released when the diaphragm moves down to the diaphragm control channel. CTCs are captured with an average 183-fold enrichment and 93.8% yield. CTC clusters, which are two or several CTCs attached to each other, are correlated with worse survival rates in patients with cancer, such as prostate, breast, colorectal, and lung cancer [30]. Thus, CTC clusters play a vital role in the metastatic process. Another filtration chip for capturing two-cell clusters (CTC clusters) was developed by Sarioglu et al. [31]. CTC clusters are captured at the edge of the triangular-shaped structure positioned inside the microfluidic channels. Captured CTC clusters are fixed in the structure by a dynamic force balance. CTCs are released by the reverse flow direction. The release efficiency of captured CTC clusters was reported as 37% at room temperature; however, the efficiency was 80% at 4 °C because lower temperatures reduce nonspecific cell adhesion. The triangular-shaped structures and the dynamic force balance prevent passing cells, while individual cells in normal filter structures can pass because of cell deformability.

Hydrodynamic microfluidic devices use inertial forces that force particles or cells to migrate across flow stream lines to equilibrium positions. In general, inertial forces emerge from boundary effects of fluid flow adjacent to the walls of a microfluidic channel [32]. Particle behavior in microfluidic devices is related to the particle and channel Reynolds number, a dimensionless number that measures the ratio of inertial forces to viscous forces. CTCs can be separated and concentrated from blood cells using the difference in inertial force, which is different for each particle, by adjusting the shape and dimensions of the channel and the flow rate [33]. Moreover, the hydrodynamic microfluidic devices can overcome obstacles associated with filtration methods such as clogging problems, low throughput, and limitation of sample volume. One inertial effect enables the inertial migration of particles. Hyun et al. developed a parallel multi-orifice flow fractionation (p-MOFF) device that consists of an alternating series of contraction channels and expansion chambers (Figure 3d) [27]. Their p-MOFF device was able to separate CTCs, which have various surface protein expression levels, from blood cells by size. Moreover, they optimized their experimental conditions for the separation of small CTCs. EMT-induced spherical cancer cells are smaller than adherent cancer cells. Separation of adherent and spherical MCF-7 cells was demonstrated by using p-MOFF, and 90.78 ± 2.21 and 80.81 ± 5.49% of adherent and spherical MCF-7 cells, respectively, were separated. CTCs from 24 metastatic breast cancer patients were isolated according to differences in EpCAM-positive and EpCAM-negative CTCs; 33.3% of patients had EpCAM-negative CTCs, and 16.7% of patients had EpCAM-positive CTCs. Another inertial effect is the secondary flow in curved channels. Larger particles in flow interrupt different secondary flow velocity fields than smaller particles [34]. The inertial fluid in spiral microfluidic channels creates secondary cross-sectional flows (Dean Flows) and causes Dean drag force, which reinforces the lateral migration of particles in the cross-section of the channel. This enables differential particle movement and results in particles in different positions according to size. In general, a spiral microfluidic channel has a rectangular cross-section. However, Shen et al. developed a dimension-confined spiral channel (D-channel) that combined a spiral microchannel and ordered micro-obstacles (bars) [35]. The D-channel assists particle focusing in order and makes streamline and main flow velocity more changeable without the need of a parallelization design or the assistance of sheath fluid. It produces more equilibrium positions for particles and increases the distances among the equilibrium positions of particles in the D-channel compared to that with a conventional spiral channel. This makes it possible to separate particles with high resolution. The separation efficiencies of particles for 15.5, 9.9, and 7.3 μm were reported as 98.7%, 97.8%, and 85.8%, respectively, with respective purities of 97.5%, 86.1%, and 98.4%. This combined microfluidic device makes a single-layer, sheathless, and ultra-low-aspect ratio microchannel system possible. However, it is hard to completely separate CTCs and WBCs because they have overlapping sizes.

### 2.3. Negative Enrichment of CTCs

**Figure 4:**
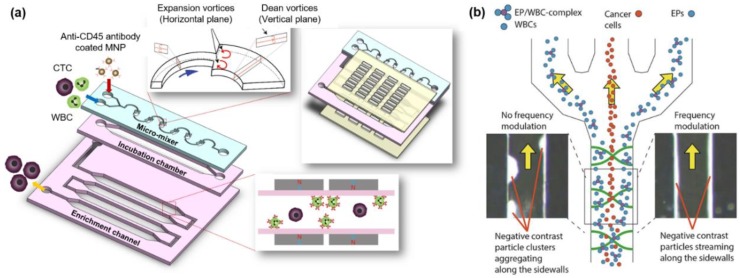
Negative enrichment of CTCs. (**a**) The micro-MixMACS chip for one-step negative enrichment of CTCs. Anti-CD45 coated magnetic nanoparticles (MNP) and WBC are bound by expansion and Dean vortices in a Micro-mixer. WBCs conjugated with MNP are captured in the enrichment channel by magnets and pure CTCs are collected in the outlet; (**b**) Separation process of the acoustophoresis chip for CTC enrichment using frequency modulation. CTCs, WBCs, and elastomeric particles (EP)/WBC-complex are injected in the acoustophoresis chip. CTCs move in the middle of the chip and others separated to the sidewall by acoustic forces. Reproduced from ref. [17,38] with permission from 2017 ELSEVIER and 2018 ELSEVIER.

Positive enrichment methods cannot isolate CTCs depending on heterogeneous properties, but negative enrichment methods can isolate heterogeneous and intact CTCs by specifically eliminating blood cells [36]. Bu et al. developed an anti-CD45 antibody-coated dual-patterned immunofiltration (DIF) device for negative enrichment of CTCs [37]. The DIF device was designed to have a dual pattern layer to increase the coating efficiency of the CD45 antibody, allowing it to improve the chance of binding leukocytes. A human non-small cell lung (NSCL) cancer cell line was used to test the performance of the DIF device. CTCs were also collected from the blood of 11 patients with cancer using the DIF device. The DIF device eliminated more than 97% of leukocytes, and fewer than 10% of CTCs were bound on the surface of the DIF device by a non-specific binding. However, as mentioned above, the method of using the binding of a cell to an antibody coated on the channel wall has a disadvantage in that the throughput is low because of the shear force. Hyun et al. combined a micro-mixer and magnetic activated cell sorter (MACS) to improve the throughput of negative enrichment (Figure 4a) [17]. The WBC-specific anti-CD45 antibody-coated magnetic particles and cells are injected into the microfluidic device. The WBCs and magnetic particles are antigen-antibody bound by the Dean drag force and expansion flow in the micromixer. The WBCs coated with magnetic particles move to the MACS channel and are captured by the magnet in the channel while the CTCs exit the channel. Regarding the performance of the micro-MixMACS, the recovery and purity of CTCs were reported as 90.67% and 0.35% in one-step. The micro-MixMACS reduced CTC loss because the magnetic particles are attached to the WBC and the separation of the CTC is performed in one step. Generally, there are ~10^7^–10^8^ WBCs per 1 mL of blood, so there is a limitation that when the WBC-coated magnetic particles are collected in the channels using magnetic force, the channels can become clogged. In particular, because CTCs are very rare, it is necessary to separate large volumes of blood to collect a sufficient number of CTCs for analysis. Therefore, the clogging issue is a major problem regarding chip continuity for high-capacity isolation. Cushing et al. developed a cancer cell separation system using negative acoustic contrast elastomeric particles (EPs) activated with CD45-antibodies (Figure 4b) [38]. They solved the clogging issue by using acoustophoresis in a continuous manner. Activated EPs and WBCs are mixed in a ratio of 1 to 30 to make EP/WBC complexes. Samples containing CTCs, Eps, and EP/WBC complexes are injected into an acoustophoresis chip (sample flow input: 100 mL/min, sheath flow input: 400 mL/min, center outlet flow: 100 mL/min, side outlet flow: 400 mL/min, frequency: 1.991 MHz with 20 V_peak-to-peak_, and scan rate: 200 kHz/ms). The performance of the acoustophoresis chip was tested at three different cell mixture conditions (1 × 10^5^ WBCs and 1 × 10^3^ MCF-7 cells in 1 mL): a mixture without EPs denoted Mixture as a control, cell mixture with non-CD45 activated EPs denoted −CD45, and cell mixture with CD45 activated EPs denoted +CD45. The separation efficiency of MCF-7 cells was reported as 98.1% for Mixture, 97.6% for −CD45, and 100% for +CD45. The recovery of MCF-7 cells was reported as 89.5% for Mixture, 93.2% for −CD45, and 86.8% for +CD45. The separation efficiency of DU145 cells was reported as 99.1% for mixture, 100% for −CD45, and 100% for +CD45. Recovery of DU145 cells was reported as 84.8% for mixture, 86.0% for −CD45, and 84.0% for +CD45. The recoveries of two different cancer cell lines are not different and are high. However, approximately 1% of sample volume still remains in the channel after isolation of CTCs. Experimental setup is complicated because experiment equipment such as a pressure driven unit and a function generator are required.

### 2.4. Integration of Enrichment Methods

**Figure 5:**
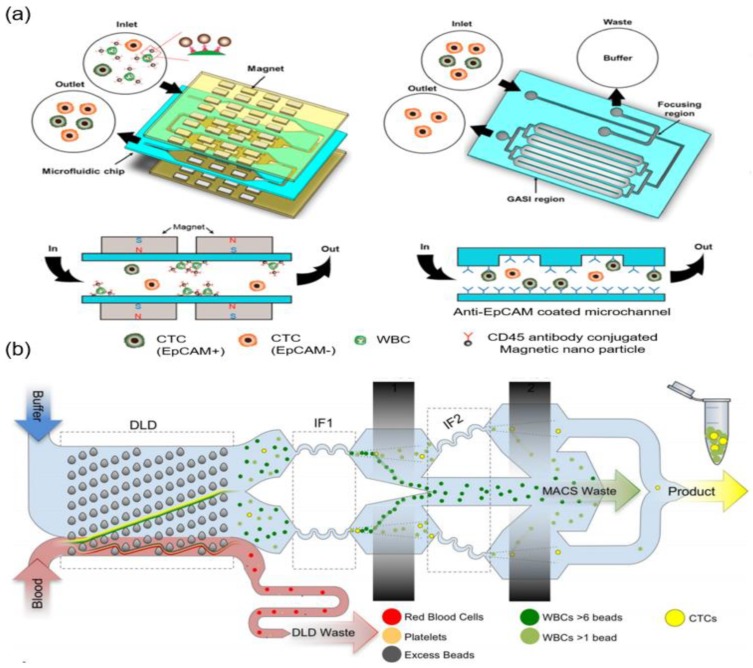
Integration of CTC enrichment methods. (**a**) Two-stage microfluidic chip consisting of a focusing region, geometrically activated surface interaction (GASI) region, and magnetic-activated cell sorter (MACS) to selectively separate CTCs depending on EpCAM expression level. Magnets in the first stage chip separate CTCs and WBCs bound with CD-45 antibody-coated magnetic particles. CTCs pass through the focusing region and move into EpCAM antibody-coated GASI region. EpCAM (+) CTCs are captured in the surface of the GASI region and EpCAM (−) CTCs are separated; (**b**) The monolithic chip integrates with deterministic lateral displacement (DLD), inertial focusing, and MACS to split purified CTCs from blood cells. Blood cells injected the monolithic chip and red blood cell (RBC) and platelets are removed in DLD region. CTCs and WBCs are aligned in the inertial focusing channel and two cells are separated in the MACS channel. Pure CTCs are collected after passing through the 2nd inertial focusing and MACS channel. Reproduced from ref. [36,39] with permission from 2015 ELSEVIER and 2017 Nature Publishing Group.

Integration of positive and negative enrichment methods is a recently emerging technology. Integrated enrichment methods refer to the use of the various CTC separation methods described above in accordance with the purpose of the study. Moreover, by combining various separation methods, the limitations of each separation method can be alleviated. Hyun et al. developed a two-stage enrichment chip that consists of a microfluidic magnetic-activated cell sorter (μ-MACS) and an inertial focusing channel combined with a focusing-geometrically activated surface interaction (F-GASI) chip (Figure 5a) [36]. WBCs coated with CD45 antibody-conjugated magnetic nanoparticles are captured by the magnet in the μ-MACS. All the isolated cells from the μ-MACS chip pass through the inertial focusing channel before moving into the GASI to reduce sample volume. In their study, EpCAM-positive cells (MCF-7) or the HER2-positive cells (SK-BR-3) were captured on the surface of the GASI. The EpCAM- and HER2-negative cells (MDA-MB-231) moved out and were collected. The capture efficiencies in the Anti-EpCAM-coated F-GASI were 98.81% of MCF-7 cells (EpCAM positive) and 93.12% of MDA-MB-231 cells (EpCAM negative). The capture efficiencies in the Anti-HER2-coated F-GASI were 86.51% of SK-BR-3 cells (HER2 positive) and 66.54% of MDA-MB-231 (HER2 negative). CTCs were selectively separated depending on differences in EpCAM and HER2 expression levels. However, operation of the chip for CTC enrichment does not work continuously because each stage microfluidic chip has a different flow rate. It creates sample loss during the experiment.

A monolithic microfluidic device that consists of DLD, inertial focusing, and MACS channels was developed by Karabacak et al. (Figure 5b) [39]. The DLD is a particle separation mechanism that uses the laminar flow characteristics of microfluidic flow in a micro-pillar array. Small particles follow the fluid flow, while large particles follow the tilted micro-pillar array at an angle to the fluid flow. In their study, whole blood that was pre-labeled with magnetic particles targeting WBCs via CD45, CD16, and CD66b surface antigens was injected to the chip. RBCs and platelets were eliminated in the DLD region. Other cells (CTCs and WBCs with few magnetic particles) moved into the inertial focusing and MACS channels. In order to increase the purity of CTCs, CTCs and WBCs were aligned in the center of the channel in the inertial focusing region to effectively remove the WBCs in the MACS channel. The percentage of CTCs after isolation from patients with cancer was 84.0% for patients with breast cancer, 68.5% for patients with lung cancer, 96.4% for patients with prostate cancer, and 63.6% for patients with melanoma. This microfluidic device enabled the depletion of blood cells at 15–20 million cells per second while isolating highly purified CTCs. The monolithic microfluidic device achieved the high-throughput negative selection of CTCs controlled by on-chip fluidic resistors.

Jiang et al. developed an integrated microfluidic device that consists of a DLD chip for isolating CTCs, an automatic purifying device with CD45-labeled immunomagnetic beads, and a capturing platform coated with rat tail collagen [40]. Rat tail collagen was used for binding CTCs tightly to the surface. Almost 100% of RBCs and platelets and over 90% of WBCs were depleted in the DLD chip. The remaining WBCs were removed by the automatic purifying device to enhance the purity of CTCs. CTCs were captured on the capturing platform. The capture rate, purity, and viability of CTCs were reported as 90%, about 50%, and more than 90%, respectively. The device is sterile, automatic, and convenient for clinical applications.

## 3. Single CTC Analysis Using Microfluidic Devices

Cancer is not a simple disease for which the outcome of treatment can be predicted or confirmed for each patient, because it is a collection of various diseases and is heterogenic in terms of genetic abnormalities and protein expression. Identifying the CTC phenotype is important in designing customized treatments for patients with cancer. However, the molecular analysis of each CTC is limited because of the heterogeneity of CTCs. In order to overcome these limitations, single cell analysis has been studied in a variety of ways such as microscopic imaging, patch clamp, flow cytometry, tweezing, patterned substrates, and microfluidic devices. Among them, the advantage of a microfluidic-based single-cell analysis system is that it can reduce experimental steps by integrating all processes from isolation to analysis continuously.

**Figure 6:**
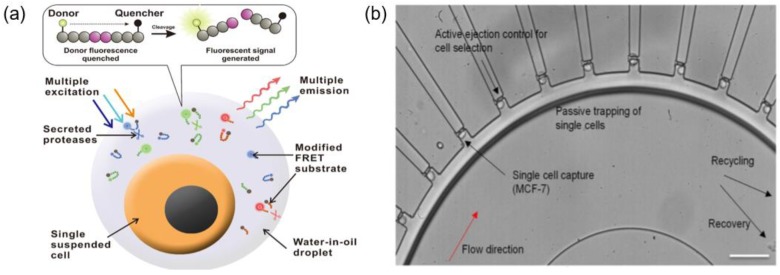
Single CTC isolation using microfluidic chip. (**a**) Generation of water-in-oil droplets in a high throughput manner (~100 cells/experimental run) that contain a single cell with Multi-color Forster Resonance Energy Transfer (FRET)-based enzymatic substrates to measure multiple protease activities specifically; (**b**) Selective picking and isolation of single CTC in each chamber could be performed by hydrodynamic focusing, thereby tracking variation after drug treatment using a model PC9 cell line. The scale bar represents 100 μm. Reproduced from ref. [42,43] with permission from 2016 ELSEVIER and 2016 Nature Publishing Group.

Droplet microfluidics is one of the most widely used methods for single cell isolation. These microfluidic devices can produce femto-liter- and pico-liter-sized aqueous droplets with high throughput on immiscible substrates such as oil. It is also one of the most promising methods to capture and analyze thousands of individual cells for whole transcriptome or genomic analysis. Cells are isolated by forming droplets in a single cell unit through microfluidic devices, and droplets containing individual cells can be used for investigating the properties of each cell, assessing cell viability, and so on. In addition, the ability to extract each target cell with high throughput can be a great advantage for single-cell screening applications, such as mutant library screening. Ben et al. generated 35 pL droplets (water in oil) using microfluidic technology, and a mixture of tumor cells from a cancer cell line (lung A549 cells) and WBCs were encapsulated according to a Poisson distribution in the presence of culture medium and a lactic acid assay mixture. Because of the differences in lactate secretion rates, there were intensity differences between droplets with a cancer cell, with a WBC, and that were empty. Each droplet can be analyzed and sorted using a laser induced fluorescence and inverted microscope with ratiometric dye (Snarf-5F). When A549 tumor cells were mixed with WBCs in ratios ranging from as few as 10:200,000 to 130:200,000 (A549 cells:WBCs), the average detection rate of A549 cells was around 60%. This system not only provides initial evidence of cancer cell metabolism but also allows the counting of CTCs in blood [41]. Ng et al. developed a droplet-based microfluidics platform to measure multiple specific protease activities from water-in-oil droplets that contained single cells (Figure 6a). By integrating the microfluidic platform with a computational analytical method, they successfully characterized six essential protease activities (MMP-2, MMP-3, MMP-9, ADAM-8, ADAM-10, and ADAM-17) in a high-throughput manner. Moreover, protease activity profiles were analyzed at single-cell level with three types of cancer cells (PC-9 lung cancer cell line, MDA-MB-231 breast cancer cell line, and K-562 leukemia cell line) [42]. The continuous isolation and identification of single CTCs from blood using droplet microfluidics have been successful, but gene analysis of individual CTCs and customized treatment through drug screening are still being developed.

On the other hand, several groups have developed novel microfluidic devices without droplets to isolate and analyze each CTC to determine the appropriate treatment for cancers. Yeo et al. developed a circular-shaped microfluidic device capable of separating a single CTC from a large population of other cells through fluid-mechanical focusing with the help of a viscous sheath flow buffer (Figure 6b). Because the cell chambers are located along the outer curvature of the circular channel, single cells are captured in each chamber due to the inherent differential pressure and centrifugal force. They successfully separated target cells using selection mechanisms such as tagging antibody markers by immunofluorescence staining, and positive pressure was exerted through a particular chamber for cell recovery. After isolating pure tumor cells from mixed populations, they tracked T790M mutations before and after drug treatment using PC9 cell lines. They enriched CTCs from 5.9 mL to 7.5 mL of blood, and 26 potential single cells were isolated from patients with late stage NSCLC (non-small cell lung cancer), and multiplex polymerase chain reaction (PCR) was performed to enrich for two sites within the EGFR gene, namely T790M and L858R [43]. Using a pancreatic cancer mouse model, Ting et al. compared the genomic expression profiles of individual CTCs isolated using an epitope-independent microfluidic device system and performed single-cell RNA sequencing. Isolation of mouse pancreatic CTCs was performed using hydrodynamic sorting and inertial focusing with magnetic separation. They obtained high-quality transcriptomes for 93 single mouse pancreatic CTCs and presented a detailed analysis of CTC composition and diversity in pancreatic cancer [44].

## 4. Critical Concerns

### 4.1. Advantages of CTC Research Compared with That Using Various Circulating Biomarkers

Circulating biomarkers obtained through liquid biopsy are an important research topic that will lead to changes in the paradigm of disease diagnosis and prognosis because of the ability to obtain patient information in real time in a less-invasive manner [45]. In addition to CTCs, as mentioned in the above sections, various circulating biomarkers have been studied regarding cancer. In particular, exosomes and cell-free DNA (cfDNA) have attracted attention as potential circulating biomarkers and are able to complement the technical hurdles in CTC studies such as rarity and heterogeneity.

Exosomes are small extracellular vesicles (30–150 nm in diameter) of endocytic origin [46]. They contain not only genetic material (double-stranded DNA (dsDNA), single-stranded DNA (ssDNA), functional messenger RNA (mRNA), microRNA (miRNA), and other small noncoding RNA molecules) and proteins (enzymes, signal transduction proteins, and cytoskeletal proteins) from their origin cell but also various proteins (tetraspanins, membrane proteins, cytokines, and cell adhesion molecules) that are present in their encapsulated lipid bilayer. With this cargo, exosomes act as intercellular communicators [47]. Currently, exosomes are also known to play an important role in the communication of cancer cells in the tumor microenvironment [48]. In this regard, exosomes can be a powerful tool for cancer diagnosis, prognosis, and personalized medicine research by analyzing the exosome cargo as well as the level of exosomes in the blood. Over the last several years, numerous studies have been published that used microfluidic technology to isolate, detect, quantify, and analyze exosomes [49]. However, the microfluidic technology has limits in dealing with exosomes because of their nanometer size. For example, inertial microfluidics, a representative microfluidic technology, is not suitable for application in studies on exosomes because the physical characteristics of particles such as size and density are of prevailing importance. In addition, whole cells in the body release exosomes during their normal metabolism. The level of exosomes in patients with cancer is known to be higher than normal because the abnormal metabolism of cancer cells. However, in order to identify the correlation between exosomes and cancer characteristics, it is necessary to determine whether exosomes are derived from cancer cells or normal cells. It has been reported that only a small fraction of exosomes out of the total exosomes per mL of blood from patients with cancer are released from cancer cells [50]. Even if the exosomes are successfully isolated from blood, the information obtained from exosome cargo is more limited than the information obtained from CTCs by the nature of their formation.

Cell-free DNA (cfDNA) is DNA that is released into the bloodstream from apoptotic, necrotic, and viable cells. When the cfDNA originates from cancer cells, it is called circulating tumor DNA (ctDNA). Up to 3.3% of tumor DNA per 100 g tumor weight is expected to enter the bloodstream daily [51]. cfDNA has various forms such as unbound DNA fragments, nucleosomes, and virtosomes, which are complexes of newly synthesized DNA, RNA, and lipoproteins [52]. This highly fragmented cfDNA consists of 150–200 base pairs and is 49.5–66 nm in length [53]. In general, cfDNA is digested by DNases in the bloodstream, but the concentration of cfDNA in patients with cancer is known to be higher than normal because cfDNA in patients with cancer is digested less because of low DNase levels and the presence of DNase inhibitors [54]. Moreover, when patients with cancer receive treatment that causes tissue damage such as surgery, chemotherapy, and radiotherapy, the concentration of cfDNA increases because of cell death and destruction. However, increased cfDNA levels are observed in patients with inflammatory diseases, tissue injuries, and benign tumors. Thus, the cfDNA level is not able to fully reflect the status of cancer. Genometastasis, in which ctDNA with an oncogenic gene is able to transform the cells of distant organs, was proposed by Garia-Olmo et al. in 1999 [55]. Several studies have reported that the transfer of DNA can occur via the uptake ctDNA by host cells, and these host cells are transformed into cancer cells. In addition, the transformation to cancer cells does not occur in the absence of ctDNA in normal plasma/serum [56]. These studies support the role of ctDNA in cancer metastasis. Thus, cfDNA should be genetically analyzed for use as a more informative biomarker. In order to avoid analysis errors in DNA sequencing, purification steps or a highly sensitive sensing system is needed because ctDNA is diluted by cfDNAs derived from normal cells. There are many commercial kits and microfluidic devices for the purification of genomic DNA. However, most of them focus on DNA purification rather than cfDNA. Because cfDNA is much shorter than genomic DNA, the experimental set-up such as the composition of the buffer to specifically purify cfDNA should be precisely controlled.

While exosomes and cfDNA can only be used in limited applications because they provide fragmented analytical information, CTCs can be used to meet a variety of research objectives. This is the main reason why CTC studies should be continued despite the rarity and heterogeneity of CTCs (Table 1). In particular, recent progress in the in vivo (patient derived tumor xenograft, PDTX) and in vitro culture of CTCs has improved the understanding of the molecular mechanisms underpinning cancer progression, depending on the conditions of an individual patient [57].

### 4.2. Commercialization of Microfluidic-Based CTC Research

The growing prevalence of cancer has raised the need for effective tools for early cancer diagnosis and application of precision medicine. CTCs are an attractive option to satisfy these needs because of their relevance to the primary tumor and the availability of liquid biopsy. Microfluidic-based products for CTC isolation and analysis based on technically mature CTC isolation methods, such as size-based filtration and immunomagnetic separation, have been commercialized (Table 2). For commercialization, the performance of the microfluidic chip is also crucial, but it is very important to develop a machine that automates the related product so that it can be used by non-experts. Unlike general purpose microfluidic chip automation machines, because of the nature of CTCs, sophisticated tubing to avoid CTC loss and complex programming to meet the needs of various users are the main factors to consider in designing automation equipment. However, these make the cost of the equipment higher, which increases the barrier for entry into the market. The market for CTC detection is expected to reach an estimated value of several billion US dollars within a few years. However, indeed, the average revenue of most microfluidic device-based companies, according to a search on D&B Hoovers (an American business research company that provides information on companies), is just 2.76 million US dollars. In order to increase the demand for products and enter into a larger market, a standard operating procedure should be established for efficient acquisition of CTCs in blood and clinical validity must be verified. Thus, extensive correlational studies and clinical trials should be conducted with CTCs and patients with cancer.

In addition, as the CTC studies continue, there are limitations due to the various features of CTCs, such as MET, in most commercialized techniques. The products based on cutting-edge technologies such as nanomaterials and multifunctional antibody are expected to overcome this issue. However, there remains realistic problems like reliability and stability to reach mass production. Also, most of the products are optimized with their own experimental conditions like working buffer for antibody conjugation and for CTC separation device. So, companies are asked to launch the related products to run their techniques. Although selling consumptive experimental materials seems to be good for profit, it takes a lot of time and money to build a manufacturing facility. Therefore, it is necessary to start the research with the aim of commercialization from the development stage. As a promising business model, some companies offer down-stream analysis after CTC enrichment and corporate with hospitals to provide clinical finding to the patients. Certifications from proven institutions may be challenging, but they will be very attractive in smaller laboratories or hospitals where it is difficult to purchase expensive devices and equipment for CTC research.

## 5. Conclusions

In this review, we described the trends in CTC research and have divided the CTC study into three generations. Before using microfluidics, the CTC isolations were processed with batch systems, but it was difficult to access a sufficient number of purified CTCs for analysis (1st generation). The various advantages of microfluidics make isolation and analysis of CTCs very efficient (2nd generation). Microfluidic based CTC isolation approaches are classified as four types: (1) Positive enrichment using antigen-antibody reaction, (2) Positive enrichment based on size, (3) Negative enrichment, and (4) Integration of enrichment methods. One of these four types of isolation methods cannot be said to be superior, but can be selected or appropriately integrated according to the purpose of each study. Also, we summarized the microfluidic based single CTC analysis after isolation. Of the various single CTC analysis approaches, the droplet based approach seems to be a very promising approach to be implemented on a one chip, from single CTC separation to analysis such as digital PCR. As the third generation of CTC research, using a microfluidic technique, it is anticipated that research on an integrated chip that isolate CTC by high-purity from the blood and then analyzes it by a single cell is expected to be active in the future. Separated CTCs can be used to study mechanisms of cancer and metastasis or to influence drug development by acquiring genetic information through CTC analysis, such as next generation sequencing (NGS). Also, the development of CTC-based PDTX with individual patient information can be an effective alternative clinical trial. These future studies on CTC will be useful for cancer prognosis and personalized medicines.

## Figures and Tables

**Table 1:** Comparison of circulating tumor cells (CTCs) and other circulating biomarkers.

Circulating Biomarkers	Features	Isolation Techniques	Applications	Reference
CTC	Cancer cells derived from tumor tissue 1–100 CTCs/mL of blood Size: 5–20 μm Heterogeneity Live information	Immuno-affinity-based methods Size/density-based methods Microfluidic-based methods	Multiple chromosome abnormalities (translocation, deletion, inversion, duplication, numerical aberration) RNA profiling Protein expression Cellular communication In vitro culture In vivo study (Patient Derived Tumor Xenograft (PDTX))	--
Exosome	Extracellular vesicles derived from cells 10^5^ exosomes/mL of blood 100 cancer-related exosomes/mL of blood Size: 30–150 nm Fixed information	Differential ultracentrifugation Polymer-based isolation methods Immuno-affinity-based methods Microfluidic-based methods	Chromosome abnormalities (translocation, deletion, inversion, duplication, numerical aberration) RNA profiling Protein expression Cellular communication	[46,47,48,49]
cfDNA	Fragmented DNA derived from cells 10–30 ng/mL of healthy blood 100 ng/mL of cancer patient blood Size: 150–200 base pair Fixed information	Affinity-based methods	Chromosome abnormalities (translocation, deletion, inversion, duplication)	[51,52,53]

**Table 2:** List of companies working on microfluidic-based CTC analysis.

Company (Country)	Product (Chip)	Highlights	Revenue *	Year Founded	Reference
Angel plc (UK)	Parsortix^TM^ system	Size-based separation: Cell trapping Possible to harvest cells or stain CTCs in cassette 100 µL to 30 mL blood sample	$0.64 million	2003	http://www.angleplc.com
ApoCell, Inc. (USA)	ApoStream^TM^	Dielectrophoresis (DEP)-based separation 50 µL to 10 mL blood sample Sample suspension collection volume: ~1.5 mL Support user prompts for step-by-step operation	$6.27 million	2004	http://www.apocell.com
Biocept, Inc. (US)	Target Selector™ platform (CEE microfluidic chip)	Immunocapture in microfluidic chip (post) Support CTC separation (5–7 days)	$3.22 million	1997	https://biocept.com
Biofluidica, Inc. (USA)	BioFluidica’s CTC System	Immunocapture in microfluidic cartridge (sinusoidal shaped) Processes 10 mL of blood in less than 30 min	$0.29 million	2012	http://www.biofluidica.com
Celsee diagnostics (USA)	Celsee PREP 400, Celsee ANALYZER	Size-based separation: Cell trapping >56,000 cell trapping well (4~8 mL of blood) ~8 micron pore 16 samples in an 8 h shift	--	2011	https://www.celsee.com
Clearbridge Biomedics (Singapore)	ClearCell^®^ FX1 system (CTChip^®^ FR)	Size-based separation: Dean Flow Fractionation (DFF) Processes 8 mL of blood in less than an hour	$0.57 million	2009	http://www.clearbridgebiomedics.com
Cynvenio Biosystems, Inc. (USA)	LiquidBiopsy^®^ Platform (ClearID^®^ Clinical Testing)	Immunomagnetic separation within a microfluidic chip Staining and cell isolation in less than 3 h Support genomic analysis of cells prepped by CleareID^®^ (10–14 days)	--		https://www.cynvenio.com
Fluxion Bioscience, Inc. (USA)	IsoFlux CTC system, IsoFlux Cytation Imager	Immunomagnetic separation within a microfluidic cartridge Up to 4 cartridges can be loaded into the instrument at one time Processes 7–10 mL of blood in less than 2 and a half hours Supports custom assays	--		https://liquidbiopsy.fluxionbio.com/
Menarini Silicon Biosystem (USA)	DEPArray™	DEP-based fluorescently labelled cell trapping in cages 300,000 DEP cages in each cartridge Image-based cell selection (single cell resolution) Enrichment and labeling steps are required before using DEPArray™	$5.58 million	1976	http://www.siliconbiosystems.com
Vortex Bioscience	VTX-1	Size and deformability-based separation: microscale vortices Processes ~8 mL of blood in less than 1 h Collects CTCs into an Eppendorf™ tube, a Petri dish, a slide chamber, or a microwell strip	--	2010	https://vortexbiosciences.com

* Search in D&B Hoovers (www.hoovers.com).

## References

[1] Vilsmaier T., Rack B., Janni W., Jeschke U., Weissenbacher T., SUCCESS Study Group (2016). Angiogenic cytokines and their influence on circulating tumour cells in sera of patients with the primary diagnosis of breast cancer before treatment. BMC Cancer.

[2] Yu M., Bardia A., Wittner B., Stott S.L., Smas M.E., Ting D.T., Isakoff S.J., Ciciliano J.C., Wells M.N., Shah A.M. (2013). Circulating breast tumor cells exhibit dynamic changes in epithelial and mesenchymal composition. Science.

[3] Miller M.C., Doyle G.V., Terstappen L.W.M.M. (2010). Significance of circulating tumor cells detected by the CellSearch system in patients with metastatic breast colorectal and prostate cancer. J. Oncol..

[4] Chen Y., Li P., Huang P.H., Xie Y., Mai J.D., Wang L., Nguyen N.T., Huang T.J. (2014). Rare cell isolation and analysis in microfluidics. Lab Chip.

[5] Gertler R., Rosenberg R., Fuehrer K., Dahm M., Nekarda H., Siewert J.R. (2003). Detection of circulating tumor cells in blood using an optimized density gradient centrifugation. Molecular Staging of Cancer.

[6] Vona G., Sabile A., Louha M., Sitruk V., Romana S., Schütze K., Capron F., Franco D., Pazzagli M., Vekemans M. (2000). Isolation by size of epithelial tumor cells: A new method for the immunomorphological and molecular characterization of circulating tumor cells. Am. J. Pathol..

[7] Tan S.J., Lakshmi R.L., Chen P., Lim W.-T., Yobas L., Lim C.T. (2010). Versatile label free biochip for the detection of circulating tumor cells from peripheral blood in cancer patients. Biosens. Bioelectron..

[8] Thege F.I., Lannin T.B., Saha T.N., Tsai S., Kochman M.L., Hollingsworth M.A., Rhim A.D., Kirby B.J. (2014). Microfluidic immunocapture of circulating pancreatic cells using parallel EpCAM and MUC1 capture: Characterization, optimization and downstream analysis. Lab Chip.

[9] Ashworth T.R. (1869). A case of cancer in which cells similar to those in the tumours were seen in the blood after death. Aust. Med. J..

[10] Ma X., Xiao Z., Li X., Wang F., Zhang J., Zhou R., Wang J., Liu L. (2014). Prognostic role of circulating tumor cells and disseminated tumor cells in patients with prostate cancer: A systematic review and meta-analysis. Tumor Biol..

[11] Gorges T.M., Tinhofer I., Drosch M., Röse L., Zollner T.M., Krahn T., von Ahsen O. (2012). Circulating tumour cells escape from EpCAM-based detection due to epithelial-to-mesenchymal transition. BMC Cancer.

[12] Chambers A.F., Groom A.C., MacDonald I.C. (2002). Metastasis: Dissemination and growth of cancer cells in metastatic sites. Nat. Rev. Cancer.

[13] Hyun K.A., Jung H.I. (2014). Advances and critical concerns with the microfluidic enrichments of circulating tumor cells. Lab Chip.

[14] Marrinucci D., Bethel K., Lazar D., Fisher J., Huynh E., Clark P., Bruce R., Nieva J., Kuhn P. (2010). Cytomorphology of circulating colorectal tumor cells: A small case series. J. Oncol..

[15] Li X., Lewis M.T., Huang J., Gutierrez C., Osborne C.K., Wu M.-F., Hilsenbeck S.G., Pavlick A., Zhang X., Chamness G.C. (2008). Intrinsic resistance of tumorigenic breast cancer cells to chemotherapy. J. Natl. Cancer Inst..

[16] Hyun K.A., Lee T.Y., Jung H.I. (2013). Negative enrichment of circulating tumor cells using a geometrically activated surface interaction chip. Anal. Chem..

[17] Lee T.Y., Hyun K.A., Jung H.I. (2017). An integrated microfluidic chip for one-step isolation of circulatingtumor cells. Sens. Actuators B Chem..

[18] Bu J., Kim Y.J., Kang Y.T., Lee T.H., Kim J., Cho Y.-H., Han S.W. (2017). Polyester fabric sheet layers functionalized with graphene oxide for sensitive isolation of circulating tumor cells. Biomaterials.

[19] Aceto N., Bardia A., Miyamoto D.T., Donaldson M.C., Wittner B.S., Spencer J.A., Yu M., Pely A., Engstrom A., Zhu H. (2014). Circulating tumor cell clusters are oligoclonal precursors of breast cancer metastasis. Cell.

[20] Jiang X., Wong K.H., Khankhel A.H., Zeinali M., Reategui E., Phillips M.J., Luo X., Aceto N., Fachin F., Hoang A.N. (2017). Microfluidic isolation of platelet-covered circulating tumor cells. Lab Chip.

[21] Lin E., Rivera-Báez L., Fouladdel S., Yoon H.J., Guthrie S., Wieger J., Deol Y., Keller E., Sahai V., Simeone D.M. (2017). High-throughput microfluidic labyrinth for the label-free isolation of circulating tumor cells. Cell Syst..

[22] Ahmed M.G., Abate M.F., Song Y., Zhu Z., Yan F., Xu Y., Wang X., Li Q., Yang C.J. (2017). Isolation, detection and antigen based profiling of circulating tumor cells using a size dictated immunocapture chip. Angew. Chem. Int. Ed..

[23] Chang C.L., Huang W., Jalal S.I., Chan B.D., Mahmood A., Shahda S., O’Neil B.H., Matei D.E., Savran C.A. (2015). Circulating tumor cell detection using a parallel flow micro-aperture chip system. Lab Chip.

[24] Kwak B., Lee J., Lee D., Lee K., Kwon O., Kang S., Kim Y. (2017). Selective isolation of magnetic nanoparticle-mediated heterogeneity subpopulation of circulating tumor cells using magnetic gradient based microfluidic system. Biosens. Bioelectron..

[25] Kwak B., Lee J., Lee J., Kim H.S., Kang S., Lee Y. (2018). Spiral shape microfluidic channel for selective isolating of heterogenic circulating tumor cells. Biosens. Bioelectron..

[26] Qin X., Park S., Duffy S.P., Matthews K., Ang R.R., Todenhöfer T., Abdi H., Azad A., Bazov J., Chi K.N. (2015). Size and deformability based separation of circulating tumor cells from castrate resistant prostate cancer patients using resettable cell traps. Lab Chip.

[27] Hyun K.A., Koo G.B., Han H., Sohn J., Choi W., Kim S.I., Jung H.I., Kim Y.S. (2016). Epithelial-to-mesenchymal transition leads to loss of EpCAM and different physical properties in circulating tumor cells from metastatic breast cancer. Oncotarget.

[28] Renier C., Pao E., Che J., Liu H.E., Lemaire C.A., Matsumoto M., Tribouler M., Srivinas S., Jeffrey S.S., Rettig M. (2017). Label-free isolation of prostate circulating tumor cells using Vortex microfluidic technology. NPJ Precis. Oncol..

[29] Gao W., Yuan H., Jing F., Wu S., Zhou H., Mao H., Jin Q., Zhao J., Cong H., Jia C. (2017). Analysis of circulating tumor cells from lung cancer patients with multiple biomarkers using high-performance size-based microfluidic chip. Oncotarget.

[30] Au S.H., Edd J., Haber D.A., Maheswaran S., Stott S.L., Toner M. (2017). Clusters of circulating tumor cells: A biophysical and technological perspective. Curr. Opin. Biomed. Eng..

[31] Sarioglu A.F., Aceto N., Kojic N., Donaldson M.C., Zeinali M., Hamza B., Engstrom A., Zhu H., Sundaresan T.K., Miyamoto D.T. (2015). A microfluidic device for label-free, physical capture of circulating tumor cell clusters. Nat. Methods.

[32] Jin C., McFaul S.M., Duffy S.P., Deng X., Tavassoli P., Black P.C., Ma H. (2014). Technologies for label-free separation of circulating tumor cells: From historical foundations to recent developments. Lab Chip.

[33] Sollier E., Go D.E., Che J., Gossett D.R., O’Byrne S., Weaver W.M., Kummer N., Rettig M., Goldman J., Nickols N. (2014). Size-selective collection of circulating tumor cells using Vortex technology. Lab Chip.

[34] Di Carlo D. (2009). Inertial microfluidics. Lab Chip.

[35] Shen S., Tian C., Li T., Xu J., Chen S.W., Tu Q., Yuan M.S., Liu W., Wang J. (2017). Spiral microchannel with ordered micro-obstacles for continuous and highly-efficient particle separation. Lab Chip.

[36] Hyun K.-A., Lee T.Y., Lee S.H., Jung H.-I. (2015). Two-stage microfluidic chip for selective isolation of circulating tumor cells (CTCs). Biosens. Bioelectron..

[37] Bu J., Kang Y.T., Kim Y.J., Cho Y.H., Chang H.J., Kim H., Moon B.I., Kim H.G. (2016). Dual-patterned immunofiltration (DIF) device for the rapid efficient negative selection of heterogeneous circulating tumor cells. Lab Chip.

[38] Cushing K., Undvall E., Ceder Y., Lilja H., Laurell T. (2018). Reducing WBC background in cancer cell separation products by negative acoustic contrast particle immuno-acoustophoresis. Anal. Chim. Acta.

[39] Fachin F., Spuhler P., Martel-Foley J.M., Edd J.F., Barber T.A., Walsh J., Karabacak M., Pai V., Yu M., Smith K. (2017). Monolithic chip for high-throughput blood cell depletion to sort rare circulating tumor cells. Sci. Rep..

[40] Jiang J., Zhao H., Shu W., Tian J., Huang Y., Song Y., Wang R., Li E., Slamon D., Hou D. (2017). An integrated microfluidic device for rapid and high-sensitivity analysis of circulating tumor cells. Sci. Rep..

[41] Ben F.D., Turetta M., Celetti G., Piruska A., Bulfoni M., Piruska A., Bulfoni M., Cesselli D., Huck W.T.S., Scoles G. (2016). A method for detecting circulating tumor cells based on the measurement of single-cell metabolism in droplet-based microfluidics. Angew. Chem..

[42] Ng E.X., Miller M.A., Jing T., Chen C. (2016). Single cell multiplexed assay for proteolytic activity using droplet microfluidics. Biosens. Bioelectron..

[43] Yeo T., Tan S.J., Lim C.L., Lau D.P.X., Chua Y.W., Kirsna S.S., Lyer G., Tan G.S., Lim T.K.H., Tan D.S.W. (2016). Microfluidic enrichment for the single cell analysis of circulating tumor cells. Sci. Rep..

[44] Ting D.T., Wittner B.S., Ligorio M., Jordan N.V., Shah A.M., Miyamoto D.T., Aceto N., Bersani F., Brannigan B.W., Xega K. (2014). Single-Cell RNA sequencing identifies extracellular matrix gene expression by Pancreatic circulating tumor cells. Cell Rep..

[45] Pantel K., Alix-Panabières C. (2013). Real-time liquid biopsy in cancer patients: Fact or fiction?. Cancer Res..

[46] Pisitkun T., Shen R.F., Knepper M.A. (2004). Identification and proteomic profiling of exosomes in human urine. Proc. Natl. Acad. Sci. USA.

[47] Panagiotara A., Markou A., Lianidou E.S., Patrinos G.P., Katsila T. (2017). Exosomes: A cancer theranostics road map. Public Health Genom..

[48] Kharaziha P., Ceder S., Li Q., Panaretakis T. (2012). Tumor cell-derived exosomes: A message in a bottle. Biochim. Biophys. Acta.

[49] Contreras-Naranjo J.C., Wu H.J., Ugaz V.M. (2017). Microfluidics for exosome isolation and analysis: Enabling liquid biopsy for personalized medicine. Lab Chip.

[50] Whiteside T.L. (2016). Tumor-derived exosomes and their role in cancer progression. Adv. Clin. Chem..

[51] Schwarzenbach H., Hoon D.S., Pantel K. (2011). Cell-free nucleic acids as biomarkers in cancer patients. Nat. Rev. Cancer.

[52] Alix-Panabières C., Pantel K. (2016). Clinical applications of circulating tumor cells and circulating tumor DNA as liquid biopsy. Cancer Discov..

[53] Dickerson R.E., Drew H.R., Conner B.N., Wing R.M., Fratini A.V., Kopka M.L. (1982). The anatomy of A-, B-, and Z-DNA. Science.

[54] Ershova E., Sergeeva V., Klimenko M., Avetisova K., Klimenko P., Kostyuk E., Veiko N., Veiko R., Izevskaya V., Kutsev S. (2017). Circulating cell-free DNA concentration and DNase I activity of peripheral blood plasma change in case of pregnancy with intrauterine growth restriction compared to normal pregnancy. Biomed. Rep..

[55] García Olmo D., García Olmo D.C., Ontañon J., Martínez E., Vallejo M. (1999). Tumor DNA circulating in the plasma might play a role in metastasis. The hypothesis of the genometastasis. Histol. Histopathol..

[56] Spindler K.L.G. (2017). Methodological, biological and clinical aspects of circulating free DNA in metastatic colorectal cancer. Acta Oncol..

[57] Hidalgo M., Amant F., Biankin A.V., Budinská E., Byrne A.T., Caldas C., Clarke R.B., de Jong S., Jonkers J., Mælandsmo G.M. (2014). Patient-derived Xenograft models: An emerging platform for translational cancer research. Cancer Discov..

